# Supplementary figures and images for: The RNA helicase DDX6 controls early mouse embryogenesis by repressing aberrant inhibition of BMP signaling through miRNA-mediated gene silencing
Source: PLoS Genet. 2022 Oct 5;18(10):e1009967. doi: 10.1371/journal.pgen.1009967 (PMC9534413; doi:10.1371/journal.pgen.1009967)

**A**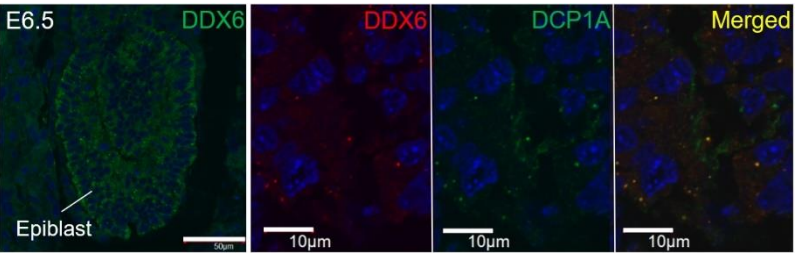**B**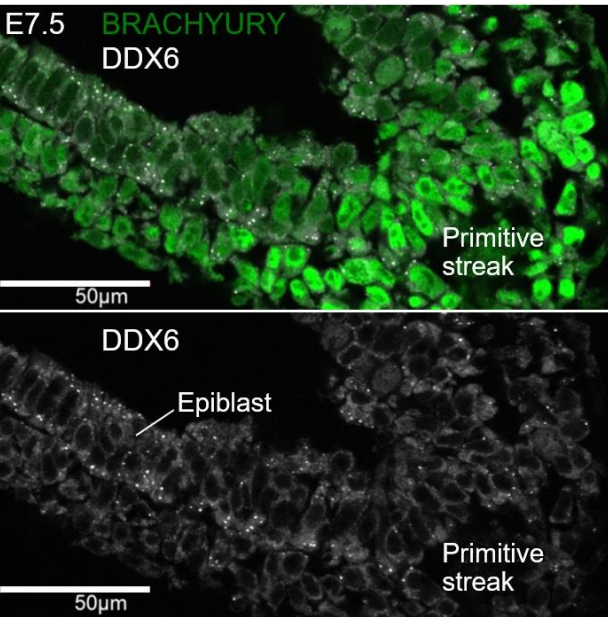**C**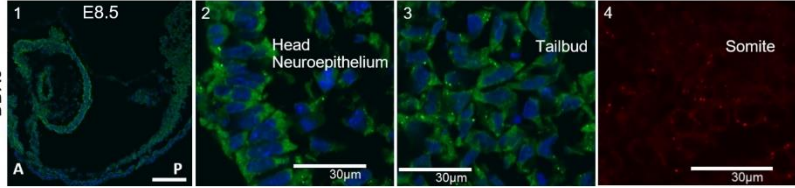**D** E8.5 *Ddx6* KO embryos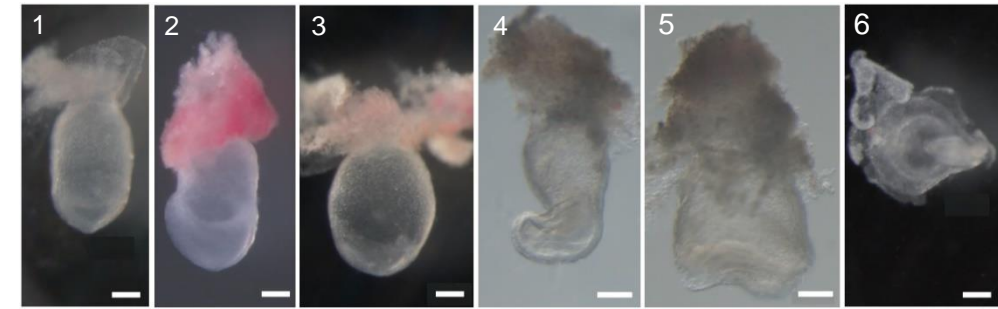**E** E9.5 *Ddx6* KO embryos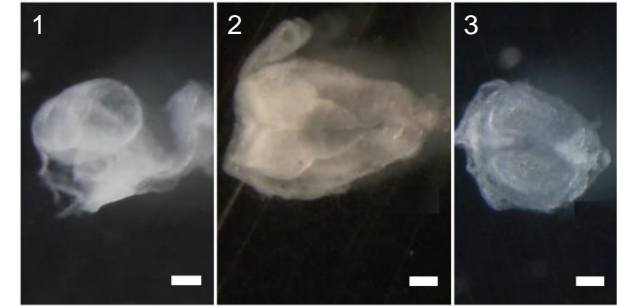**F** *Brachyury* E8.5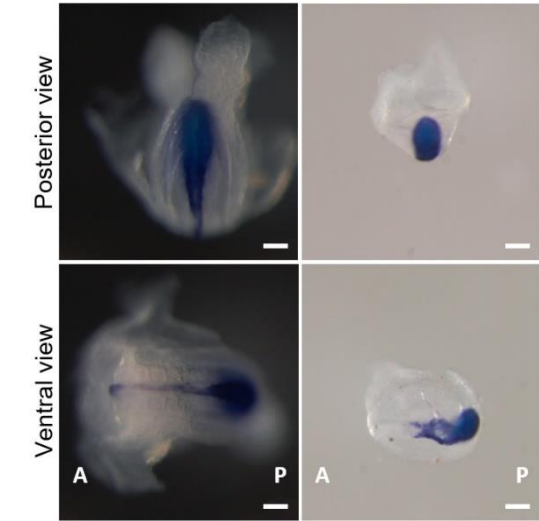

Supplement: S1 Fig — (A) E6.5 embryo frozen section IHC for DDX6 and DCP1A (Scale: 50 μm for lower magnification, 10 μm for higher magnification). DAPI in blue. (B) E7.5 embryo frozen section IHC for BRACHYURY & DDX6 (Scale: 50 μm). (C) (1–3) E8.5 embryo frozen section IHC for DDX6. (4) Image of an E8.5 embryo expressing DDX6-mCherry (Scale: 100 μm for 1; 30 μm for 2–4). DAPI in blue. (D) Variation in the morphology of E8.5 Ddx6 KO embryos. (1–3) Lateral view of embryos that were younger than the head-fold stage. (4) An embryo with a markedly severe phenotype. It lacks the entire mesoderm. (5–6) Embryos with a head-fold structure and shortened primitive streak. (Scale: 200 μm). (E) Variability of E9.5 Ddx6 KO embryos. (1) lateral view. (2–3) dorsal view. (Scale: 200 μm). (F) Whole-mount ISH of E8.5 embryos with a Brachyury probe (Scale: 100 μm). Representative images of the posterior and ventral view of the embryos, which were not shown in Fig 2A. (PDF) [file pgen.1009967.s001.pdf]

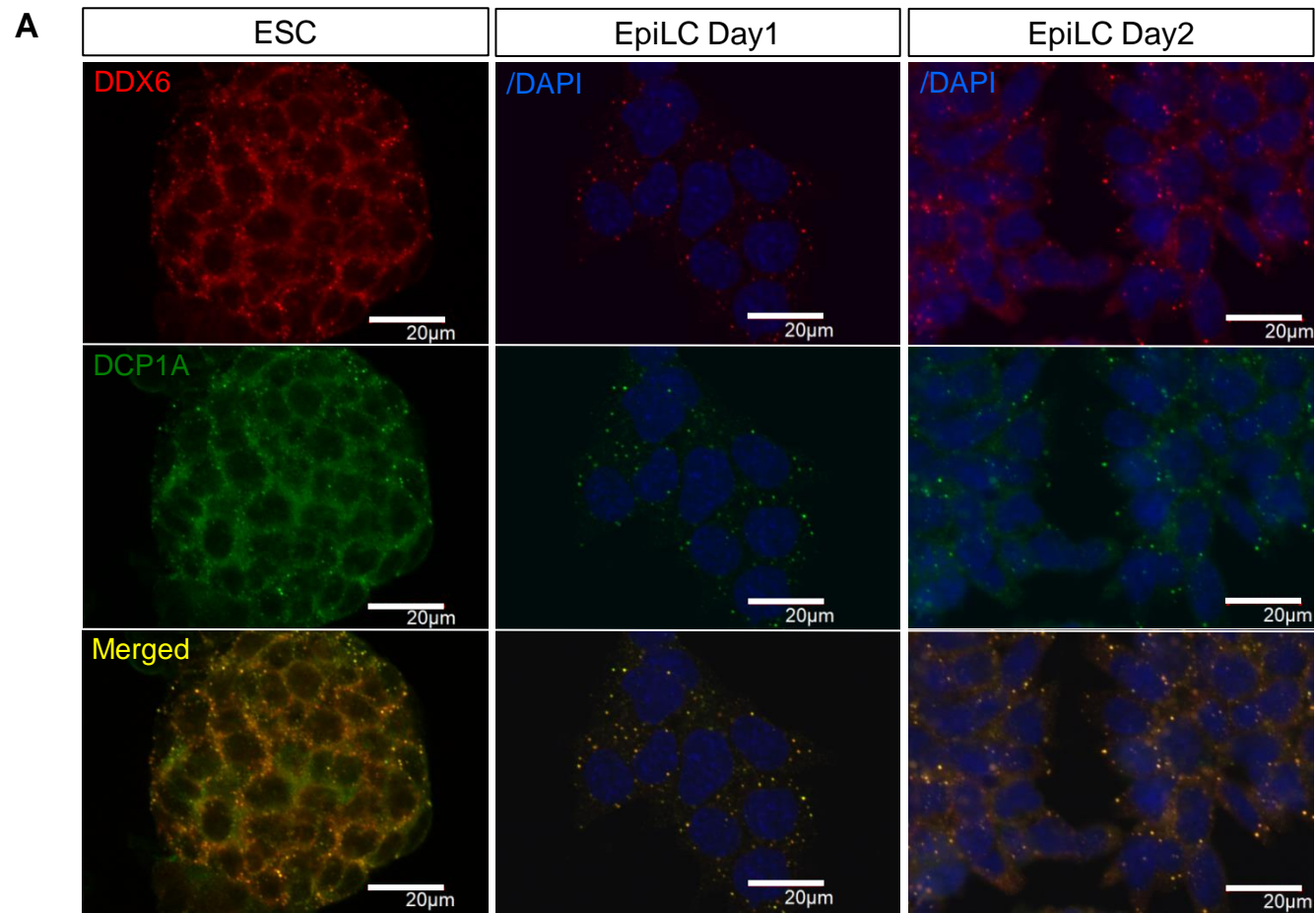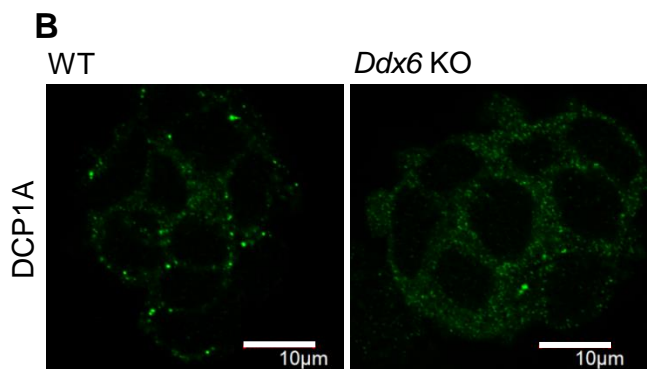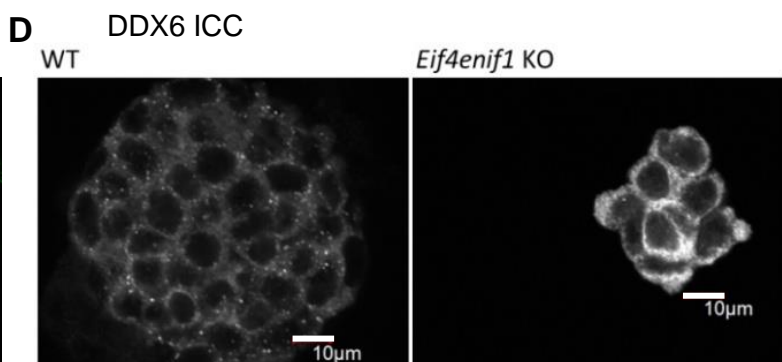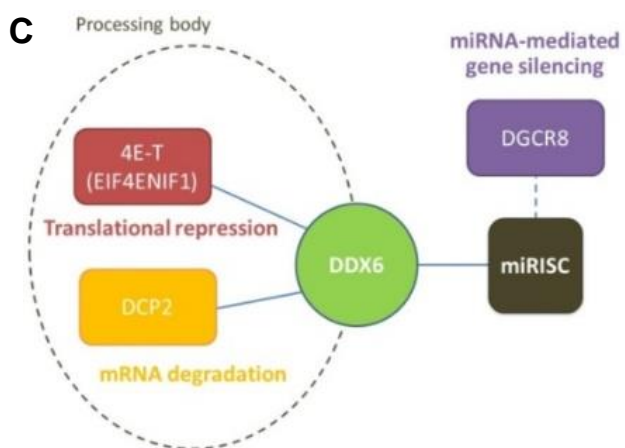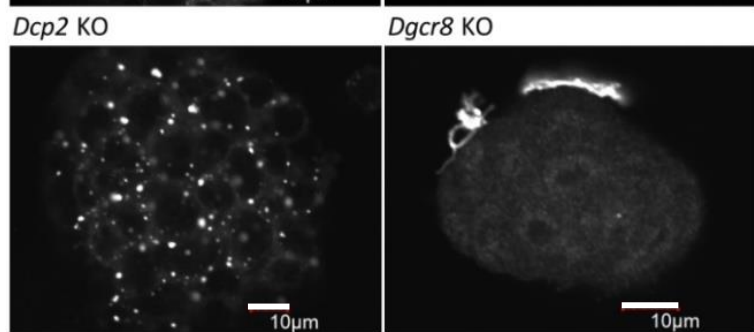

Supplement: S3 Fig — (A) ICC of DDX6 and the P-body marker DCP1A during the ESC-to-EpiLC induction period (Scale: 20 μm). (B) Distinct granular P-bodies disappeared in the absence of DDX6. ICC of DCP1A, a P-body marker, in ESCs (Scale: 10 μm). (C) A scheme of genetic dissection of the DDX6-mediated RNA regulatory pathways. Three major DDX6-mediated pathways were disrupted by knocking out the key gene of each pathway. (D) P-bodies in ESCs were affected by the deletion of each gene. ICC of DDX6, a P-body marker, in ESCs (Scale: 10 μm). (PDF) [file pgen.1009967.s003.pdf]

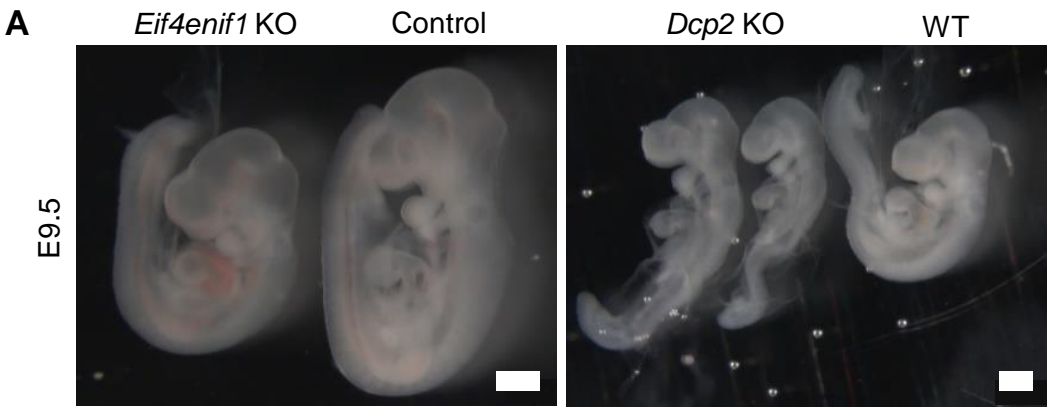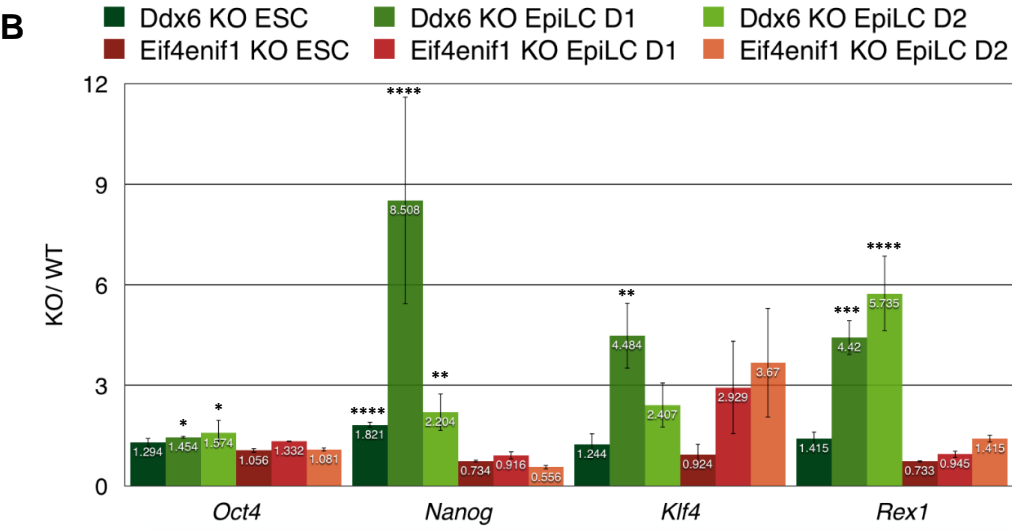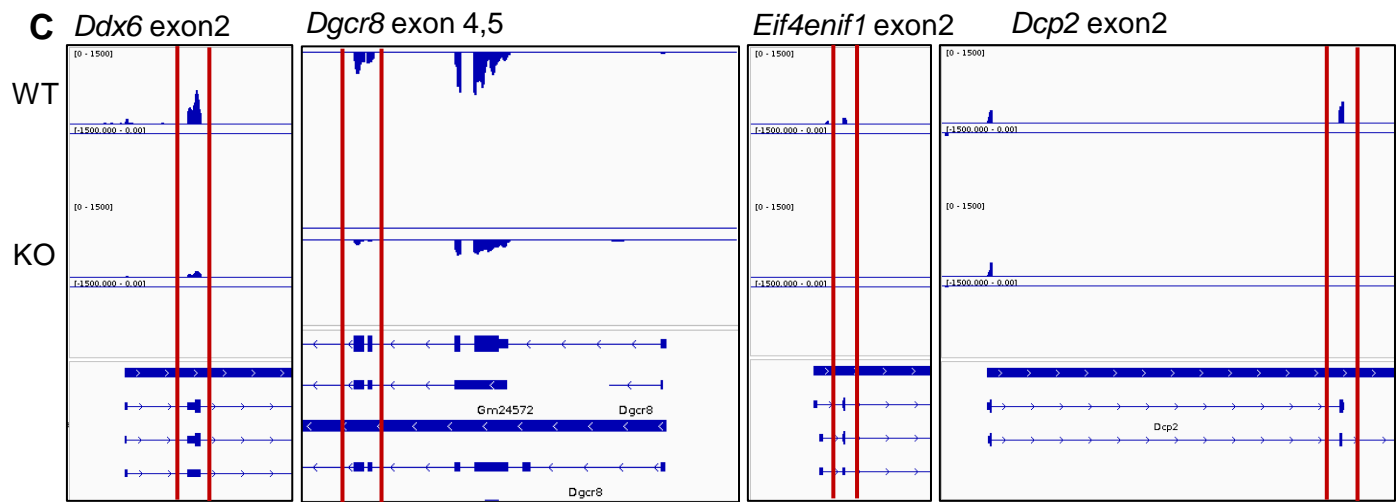

Supplement: S5 Fig — (A) E9.5 Eif4enif1 KO (Scale: 500 μm, n = 7) and Dcp2 KO (Scale: 500 μm, n = 3) embryos with a littermate control. (B) Comparison of gene expression between Ddx6 KO and Eif4enif1 KO. qRT-PCR analysis of pluripotency genes during the EpiLC induction period. Each bar represents the relative expression of KO cells to WT cells at the indicated time point. Mean ± SEM. Student’s t-test (n ≥ 3) (*p ≤ 0.05, **p ≤ 0.01, ***p ≤ 0.001, ****p ≤ 0.0001). (C) Integrative Genomics Viewer (IGV) snapshot of each locus to confirm successful targeting. (PDF) [file pgen.1009967.s005.pdf]
