# Supplementary material for: The RNA helicase DDX6 controls early mouse embryogenesis by repressing aberrant inhibition of BMP signaling through miRNA-mediated gene silencing
Source: PLoS Genet. 2022 Oct 5;18(10):e1009967. doi: 10.1371/journal.pgen.1009967 (PMC9534413; doi:10.1371/journal.pgen.1009967)
Supplement: S2 Fig — (A) Gene ontology (GO) term enrichment analysis of the most upregulated and downregulated genes. (B) RNA-seq data comparing expression of negative regulators of the BMP pathway in E8.5 Ddx6 KOs with that in E8.5 WT. (C) Classification of types of the upregulated negative regulators of BMP signaling. (D-G, I-J) RNA-seq data comparing the expression of several key genes in E8.5 Ddx6 KOs with that in E8.5 WT. (D) NSC and radial glial cell (NPC) markers. (E) Genes related to neuron-restricted intermediate progenitors and differentiated neurons. (F) Primitive streak and early mesoderm-related genes. (G) Differentiated mesoderm and endoderm markers. PSM: paraxial mesoderm, LPM: lateral plate mesoderm, DE: definitive endoderm. (I) Pluripotency marker genes. (J) BMP signaling components. (H) qRT-PCR analysis of some key genes in Ddx6 KO E8.5 embryos. Most embryos used for this analysis were younger than the head-fold stage. Mean ± SEM. Significance was calculated by the Wilcoxon rank-sum nonparametric test (n = 10, 8, 12, 12, 8, 9, 10, 9, 8, 13, 8, 10, 5, and 7 in order of genes listed) (* at the α = 0.05 significance level, ** α = 0.01). (PDF) [file pgen.1009967.s002.pdf]

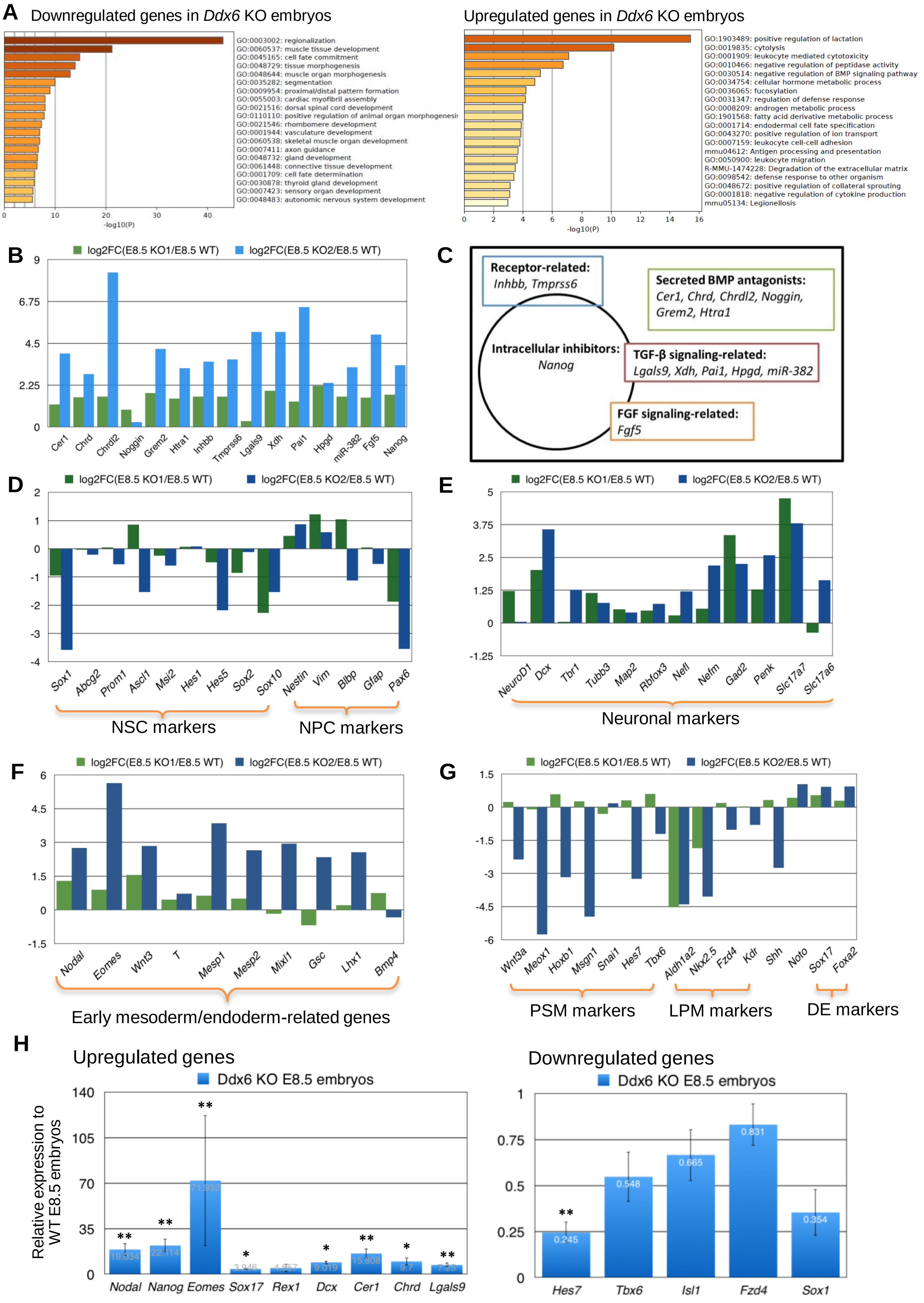
A
Downregulated genes in Ddx6 KO embryos
GO:0003002: regionalization
GO:0060537: muscle tissue development
GO:0045165: cell fate commitment
GO:0048729: tissue morphogenesis
GO:0048644: muscle organ morphogenesis
GO:0035282: segmentation
GO:0009954: proximal/distal pattern formation
GO:0055003: cardiac myofibril assembly
GO:0021516: dorsal spinal cord development
GO:0110110: positive regulation of animal organ morphogenesis
GO:0021546: rhombomere development
GO:0001944: vasculature development
GO:0060538: skeletal muscle organ development
GO:0007411: axon guidance
GO:0048732: gland development
GO:0061448: connective tissue development
GO:0001709: cell fate determination
GO:0030878: thyroid gland development
GO:0007423: sensory organ development
GO:0048483: autonomic nervous system development
-log10(P)
Upregulated genes in Ddx6 KO embryos
GO:1903489: positive regulation of lactation
GO:0019835: cytolysis
GO:0001909: leukocyte mediated cytotoxicity
GO:0010466: negative regulation of peptidase activity
GO:0030514: negative regulation of BMP signaling pathway
GO:0034754: cellular hormone metabolic process
GO:0036065: fucosylation
GO:0031347: regulation of defense response
GO:0008209: androgen metabolic process
GO:1901568: fatty acid derivative metabolic process
GO:0001714: endodermal cell fate specification
GO:0043270: positive regulation of ion transport
GO:0007159: leukocyte cell-cell adhesion
mmu04612: Antigen processing and presentation
GO:0050900: leukocyte migration
R-MMU-1474228: Degradation of the extracellular matrix
GO:0098542: defense response to other organism
GO:0048672: positive regulation of collateral sprouting
GO:0001818: negative regulation of cytokine production
mmu05134: Legionellosis
-log10(P)
B
log2FC(E8.5 KO1/E8.5 WT)
log2FC(E8.5 KO2/E8.5 WT)
Cer1 Chrd Chrdl2 Noggin Grem2 Htra1 Inhbb Tmprss6 Lgals9 Xdh Pai1 Hpgd miR-382 Fgf5 Nanog
C
Receptor-related: Inhbb, Tmprss6
Secreted BMP antagonists: Cer1, Chrd, Chrdl2, Noggin, Grem2, Htra1
Intracellular inhibitors: Nanog
TGF-β signaling-related: Lgals9, Xdh, Pai1, Hpgd, miR-382
FGF signaling-related: Fgf5
D
log2FC(E8.5 KO1/E8.5 WT)
log2FC(E8.5 KO2/E8.5 WT)
Sox1 Abcg2 Prom1 Ascl1 Msi2 Hes1 Hes5 Sox2 Sox10 Nestin Vim Blbp Gfap Pax6
NSC markers
NPC markers
E
log2FC(E8.5 KO1/E8.5 WT)
log2FC(E8.5 KO2/E8.5 WT)
NeuroD1 Dcx Tbr1 Tubb3 Map2 Rbfox3 Nefl Nefm Gad2 Penk Slc17a7 Slc17a6
Neuronal markers
F
log2FC(E8.5 KO1/E8.5 WT)
log2FC(E8.5 KO2/E8.5 WT)
Nodal Eomes Wnt3 T Mesp1 Mesp2 Mixl1 Gsc Lhx1 Bmp4
Early mesoderm/endoderm-related genes
G
log2FC(E8.5 KO1/E8.5 WT)
log2FC(E8.5 KO2/E8.5 WT)
Wnt3a Meox1 Hoxb1 Msgn1 Snai1 Hes7 Tbx6 Aldh1a2 Nkx2.5 Fzd4 Kdr Shh Noto Sox17 Foxa2
PSM markers
LPM markers
DE markers
H
Upregulated genes
Ddx6 KO E8.5 embryos
Relative expression to WT E8.5 embryos
19.034 22.114 71.932 3.946 4.657 9.019 15.808 9.7 7.25
Nodal Nanog Eomes Sox17 Rex1 Dcx Cer1 Chrd Lgals9
Downregulated genes
Ddx6 KO E8.5 embryos
0.245 0.548 0.665 0.831 0.354
Hes7 Tbx6 Isl1 Fzd4 Sox1

I

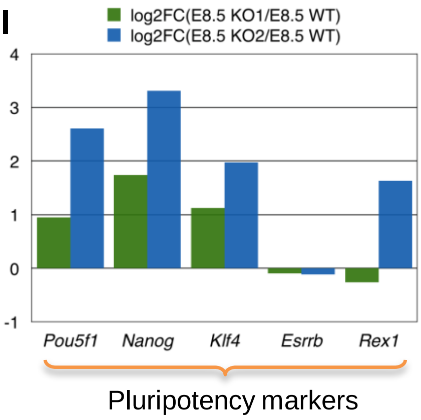
log2FC(E8.5 KO1/E8.5 WT)
log2FC(E8.5 KO2/E8.5 WT)
4
3
2
1
0
-1
Pou5f1
Nanog
Klf4
Esrrb
Rex1
Pluripotency markers

J

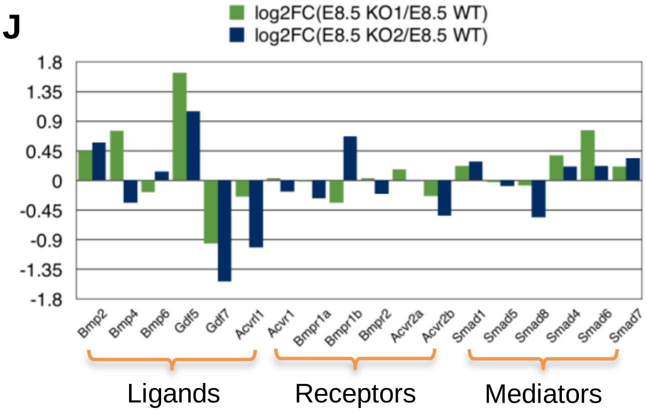
log2FC(E8.5 KO1/E8.5 WT)
log2FC(E8.5 KO2/E8.5 WT)
1.8
1.35
0.9
0.45
0
-0.45
-0.9
-1.35
-1.8
Bmp2
Bmp4
Bmp6
Gdf5
Gdf7
Acvrl1
Acvr1
Bmpr1a
Bmpr1b
Bmpr2
Acvr2a
Acvr2b
Smad1
Smad5
Smad8
Smad4
Smad6
Smad7
Ligands
Receptors
Mediators
