# Supplementary material for: The RNA helicase DDX6 controls early mouse embryogenesis by repressing aberrant inhibition of BMP signaling through miRNA-mediated gene silencing
Source: PLoS Genet. 2022 Oct 5;18(10):e1009967. doi: 10.1371/journal.pgen.1009967 (PMC9534413; doi:10.1371/journal.pgen.1009967)
Supplement: S1 Table — (PDF) [file pgen.1009967.s007.pdf]

S1 Table. Gene sets that are highly enriched in three different conditions (Top11~20)

Common in only *Ddx6* KO & *Dgcr8* KO ESCs (regulated by miRNAs/ P-body-independent)	Common in all four KO ESCs (regulated by miRNAs/P-body-dependent)	Common in only *Eif4enif1* KO & *Dcp2* KO ESCs (regulated by P-body functions/miRNA-independent)
Upregulated gene sets Top11~Top20		
11.Regulation of extracellular matrix organization<br>12.Mononuclear cell migration<br>13.Regulation of protein maturation<br>14.Positive regulation of smooth muscle cell migration<br>15.Peptidase regulator activity<br>16.Positive regulation of epithelial cell migration<br>17.Tissue remodeling<br>18.Positive regulation of inflammatory response<br>19.Positive regulation of vasculature development<br>20.Skeletal system development	11.Extracellular matrix structural constituent conferring tensile strength<br>12.Extracellular matrix disassembly<br>13.Integrin binding<br>14.Aminoglycan catabolic process<br>15.Growth factor binding<br>16.Protease binding<br>17.Collagen metabolic process<br>18.Metalloendopeptidase activity<br>19.Cellular response to vascular endothelial growth factor stimulus<br>20.Carbohydrate binding	
Downregulated gene sets Top11~Top20		
11.Large ribosomal subunit<br>12.Organellar ribosome<br>13.mRNA transport<br>14.Establishment of RNA localization<br>15.Precatalytic spliceosome<br>16.Cotranslational protein targeting to membrane<br>17.Ribosome assembly<br>18.Maturation of 5 8s rRNA<br>19.Telomere organization<br>20.Nuclear export	11.Spliceosomal complex<br>12.RNA export from nucleus<br>13.SM-like protein family complex<br>14.Small nuclear ribonucleoprotein complex<br>15.DNA templated transcription elongation<br>16.U2 Type spliceosomal complex<br>17.Translational initiation<br>18.DNA-dependent DNA replication<br>19.Cytoplasmic translation<br>20.Translation regulator activity nucleic acid binding	11.DNA binding transcription activator activity<br>12.Positive regulation of neurogenesis
