# Supplementary material for: The RNA helicase DDX6 controls early mouse embryogenesis by repressing aberrant inhibition of BMP signaling through miRNA-mediated gene silencing
Source: PLoS Genet. 2022 Oct 5;18(10):e1009967. doi: 10.1371/journal.pgen.1009967 (PMC9534413; doi:10.1371/journal.pgen.1009967)
Supplement: S2 Table — (PDF) [file pgen.1009967.s008.pdf]

S2 Table. Gene sets that are differentially expressed only in *Ddx6* KO ESCs

Differentially expressed only in *Ddx6* KO ESCs (DDX6 function that may be not associated with any of miRNAs (DGCR8), 4E-T, DCP2, and P-bodies)
Upregulated gene sets Top20
1.Regulation of neuron migration<br>2.MYD88-independent toll like receptor signaling pathway<br>3.Regulation of extracellular matrix assembly<br>4.Bone growth<br>5.Negative regulation of biomineralization<br>6.Hyaluronan catabolic process<br>7.Positive regulation of small GTPase-mediated signal transduction<br>8.Negative regulation of chondrocyte differentiation<br>9.Metallocarboxypeptidase activity<br>10.Negative regulation of cartilage development<br>11.Myeloid dendritic cell activation<br>12.Positive regulation of endothelial cell apoptotic process<br>13.Calcium ion import into cytosol<br>14.Positive regulation of chondrocyte differentiation<br>15.Actin binding<br>16.FAD binding<br>17.Oxidoreductase activity: acting on the CH, NH2 group of donors<br>18.Positive regulation of leukocyte apoptotic process<br>19.Regulation of endocrine process<br>20.Actin-dependent ATPase activity
Downregulated gene sets Top20
1.Protein localization to endoplasmic reticulum<br>2.Protein targeting to membrane<br>3.Purine nucleoside monophosphate biosynthetic process<br>4.Pyrimidine nucleotide metabolic process<br>5.Pyrimidine nucleoside triphosphate metabolic process<br>6.Pyrimidine containing compound metabolic process<br>7.Pyrimidine nucleoside triphosphate biosynthetic process<br>8.Negative regulation of ubiquitin protein transferase activity<br>9.Leukotriene biosynthetic process<br>10.Pyrimidine ribonucleotide biosynthetic process<br>11.Purine nucleoside biosynthetic process<br>12.Ribosomal small subunit assembly<br>13.Alpha amino acid metabolic process<br>14.Peptidyl arginine modification<br>15.Negative regulation of cell cycle G1-S phase transition<br>16.Establishment of protein localization to membrane<br>17.Glutamine metabolic process<br>18.Pyrimidine nucleobase metabolic process<br>19.Protein targeting<br>20.Iron sulfur cluster assembly
