# Supplementary material for: The RNA helicase DDX6 controls early mouse embryogenesis by repressing aberrant inhibition of BMP signaling through miRNA-mediated gene silencing
Source: PLoS Genet. 2022 Oct 5;18(10):e1009967. doi: 10.1371/journal.pgen.1009967 (PMC9534413; doi:10.1371/journal.pgen.1009967)
Supplement: S3 Table — (PDF) [file pgen.1009967.s009.pdf]

S3 Table. Gene sets that are differentially expressed only in *Dgcr8* KO ESCs

Differentially expressed only in *Dgcr8* KO ESCs (miRNA-mediated gene regulation that may be not associated with any of DDX6, 4E-T, DCP2, and P-bodies)
Upregulated gene sets Top20
1. Polypeptide N-acetylgalactosaminyltransferase activity<br>2. Regulation of metallopeptidase activity<br>3. RNA binding involved in posttranscriptional gene silencing<br>4. Monocarboxylic acid catabolic process<br>5. Regulation of macrophage-derived foam cell differentiation<br>6. Bone resorption<br>7. Iron ion transmembrane transport<br>8. Regulation of macrophage migration<br>9. Calcium-mediated signaling<br>10. Foam cell differentiation<br>11. Positive regulation of p38 MAPK cascade<br>12. Positive regulation of macrophage migration<br>13. Positive regulation of tyrosine phosphorylation of STAT protein<br>14. Midbrain dopaminergic neuron differentiation<br>15. Antigen processing and presentation of endogenous peptide antigen<br>16. Regulation of reactive oxygen species biosynthetic process<br>17. Positive regulation of lipid catabolic process<br>18. p38 MAPK cascade<br>19. Intramembrane lipid transporter activity<br>20. C21 steroid hormone biosynthetic process
Downregulated gene sets Top20
1. Positive regulation of RNA splicing<br>2. Histone H3K4 methylation<br>3. RNA polymerase II general transcription initiation<br>4. Exon-exon junction complex<br>5. Transcription by RNA polymerase III<br>6. DNA replication checkpoint<br>7. Peptidyl lysine methylation<br>8. Oxidoreductase activity, acting on NAD(P)H, quinone or similar compound as acceptor<br>9. Proteasome accessory complex<br>10. Attachment of mitotic spindle microtubules to kinetochore<br>11. rRNA catabolic process<br>12. Peptide lysine N-acetyltransferase activity<br>13. Regulation of nucleobase-containing compound transport<br>14. Transcription initiation from RNA polymerase II promoter<br>15. Regulation of mRNA 3'-end processing<br>16. β-catenin TCF complex assembly<br>17. Blastocyst growth<br>18. Pseudouridine synthesis<br>19. Site of DNA damage<br>20. Positive regulation of chromatin organization
