# Supplementary material for: The RNA helicase DDX6 controls early mouse embryogenesis by repressing aberrant inhibition of BMP signaling through miRNA-mediated gene silencing
Source: PLoS Genet. 2022 Oct 5;18(10):e1009967. doi: 10.1371/journal.pgen.1009967 (PMC9534413; doi:10.1371/journal.pgen.1009967)
Supplement: S4 Table — (PDF) [file pgen.1009967.s010.pdf]

S4 Table. Gene sets that are differentially expressed only in *Eif4enif1* KO ESCs

**Differentially expressed only in *Eif4enif1* KO ESCs (4E-T function that may be not associated with any of DDX6, miRNAs, DCP2, and P-bodies)**
Upregulated gene sets Top20
1. Ethanol metabolic process<br>2. Organic acid transmembrane transport<br>3. Retinoic acid metabolic process<br>4. Organic acid transport<br>5. Leukotriene biosynthetic process<br>6. Oxidoreductase activity acting on peroxide as acceptor<br>7. Hexose transmembrane transporter activity<br>8. Myoblast fusion<br>9. Leukotriene metabolic process<br>10. Symporter activity<br>11. Aldehyde dehydrogenase NAD(P)+ activity<br>12. Secondary active transmembrane transporter activity<br>13. Carboxylic acid transport<br>14. Amino acid transmembrane transport<br>15. Carboxylic acid binding<br>16. Solute anion antiporter activity<br>17. Fatty acid transport<br>18. Amino acid import<br>19. Import across plasma membrane<br>20. Anion transmembrane transporter activity
Downregulated gene sets Top20
1. Pericentriolar material<br>2. Positive regulation of protein depolymerization<br>3. Genetic imprinting<br>4. Microtubule nucleation<br>5. γ-tubulin complex<br>6. Steroid hormone receptor activity<br>7. Protein demethylase activity<br>8. Hematopoietic progenitor cell differentiation<br>9. Neural tube patterning<br>10. Phosphatidylinositol 3-kinase (PI3K) complex<br>11. Negative regulation of cellular response to insulin stimulus<br>12. Response to nerve growth factor<br>13. β-tubulin binding<br>14. Fc receptor signaling pathway<br>15. Guanyl nucleotide exchange factor activity<br>16. Regulation of insulin receptor signaling pathway<br>17. Fc epsilon receptor signaling pathway<br>18. Regulation of microtubule-based process<br>19. Macromolecule deacylation<br>20. Ubiquitin-like protein transferase activity
