# Supplementary material for: The RNA helicase DDX6 controls early mouse embryogenesis by repressing aberrant inhibition of BMP signaling through miRNA-mediated gene silencing
Source: PLoS Genet. 2022 Oct 5;18(10):e1009967. doi: 10.1371/journal.pgen.1009967 (PMC9534413; doi:10.1371/journal.pgen.1009967)
Supplement: S5 Table — (PDF) [file pgen.1009967.s011.pdf]

S5 Table. Gene sets that are differentially expressed only in *Dcp2* KO ESCs

Differentially expressed only in *Dcp2* KO ESCs (DCP2 function that may be not associated with any of DDX6, miRNAs, 4E-T, and P-bodies)
Upregulated gene sets Top20
1. Endoplasmic reticulum protein-containing complex<br>2. Protein disulfide isomerase activity<br>3. Protein activation cascade<br>4. Protein N-linked glycosylation<br>5. Triglyceride-rich plasma lipoprotein particle<br>6. Peptidyl asparagine modification<br>7. Endoplasmic reticulum unfolded protein response<br>8. Intrinsic component of endoplasmic reticulum membrane<br>9. Cornification<br>10. Protein-lipid complex<br>11. Chaperone binding<br>12. Glycosylation<br>13. Sterol transfer activity<br>14. Response to endoplasmic reticulum stress<br>15. Receptor catabolic process<br>16. Spectrin binding<br>17. Oxidoreductase activity acting on a sulfur group of donors<br>18. Intermembrane lipid transfer<br>19. Lipid transfer activity<br>20. Disulfide oxidoreductase activity
Downregulated gene sets Top20
1. Central nervous system projection neuron axonogenesis<br>2. Segment specification<br>3. Roof of mouth development<br>4. Secondary palate development<br>5. Inhibitory synapse assembly<br>6. Proximal-distal pattern formation<br>7. Anterior-posterior axis specification<br>8. Regulation of cardiac muscle cell differentiation<br>9. Type B pancreatic cell development<br>10. Central nervous system neuron axonogenesis<br>11. Negative regulation of interleukin-10 production<br>12. Embryonic hindlimb morphogenesis<br>13. Trachea development<br>14. Tripartite regional subdivision<br>15. Glomerular epithelial cell differentiation<br>16. Enteroendocrine cell differentiation<br>17. Maintenance of synapse structure<br>18. Embryonic axis specification<br>19. Regulation of muscle organ development<br>20. Positive regulation of microtubule polymerization or depolymerization
