# Supplementary material for: The RNA helicase DDX6 controls early mouse embryogenesis by repressing aberrant inhibition of BMP signaling through miRNA-mediated gene silencing
Source: PLoS Genet. 2022 Oct 5;18(10):e1009967. doi: 10.1371/journal.pgen.1009967 (PMC9534413; doi:10.1371/journal.pgen.1009967)
Supplement: S4 Fig — (A) Western blot of endogenous SMAD2/3 and phosphorylated SMAD2 in mESCs. Four conditions: SB (SB431542: TGF-β receptor ALK5 inhibitor)/—(non-treated)/ Activin 1-hr treatment/ Activin 2-hr treatment. The quantified signal intensity of the band is displayed in the right-side graph (n = 8). (B) Venn diagram showing the number of peaks bound by SMAD3 and SMAD2 from ChIP-seq performed for embryoid bodies [117]. (C) Binding score of MACS2 & STRING for SMAD3 and SMAD2 to genes of interest. Mean (± SEM). (PDF) [file pgen.1009967.s004.pdf]

A

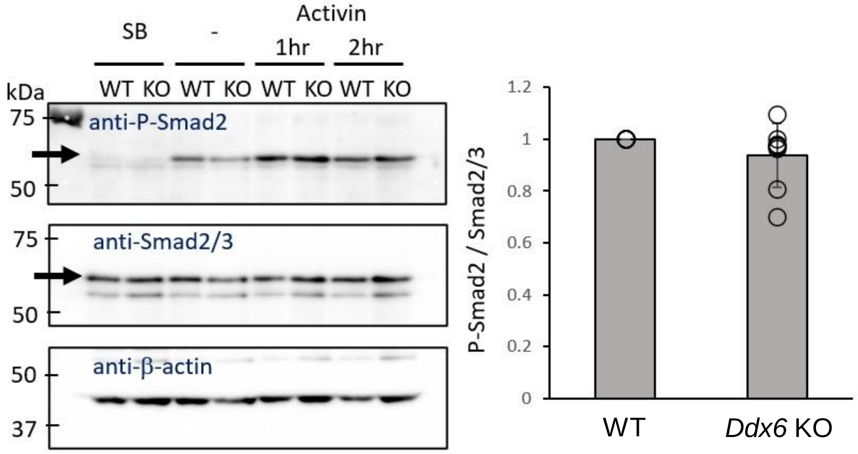

B

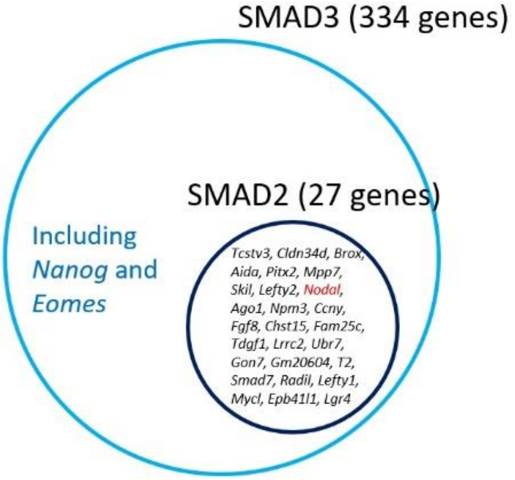

C

Color legends (Values = Binding score of MACS2 & STRING)

1000	750	500	250	1	0

	SMAD3	SMAD2
Nanog	889.5 (± 638)	619.25 (± 183)
Eomes	807 (± 51)	0
Nodal	1162 (± 869)	1161.625 (± 298)
Average in EBs	240.841	49.401
