# Supplementary material for: The RNA helicase DDX6 controls early mouse embryogenesis by repressing aberrant inhibition of BMP signaling through miRNA-mediated gene silencing
Source: PLoS Genet. 2022 Oct 5;18(10):e1009967. doi: 10.1371/journal.pgen.1009967 (PMC9534413; doi:10.1371/journal.pgen.1009967)
Supplement: Attachment — Submitted filename: Response to reviewers.docx [file pgen.1009967.s012.docx]

Responses to reviewer’s comments

**[Reviewer #1]**

General thoughts:

1) The molecular similarity between DDX6 and DGCR8 knockout ES/EpiLCs cells is quite convincing and consistent with the previous work (Freimer et al., eLIFE. 2018). The in vivo analysis is also suggestive of similarities between the two mutants, but is very limited in terms of the characterization of the DGCR8 KO embryos.

: We newly prepared more *Dgcr8* KO embryos and conducted whole-mount ISH with probes for *Brachyury, Nodal*, and *Eomes*. *Nodal* and *Eomes* were abnormally maintained in the posterior body, similar to *Ddx6* mutants. However, unlike *Ddx6* KO, which exhibited delayed primitive streak induction, *Dgcr8* KO embryos had relatively normal and strong *Brachyury* induction at E7.5 despite their small body size. However, their development was markedly disrupted during gastrulation and they had severely malformed morphology at E8.5.

2) Showing that alternative mutants of P body biogenesis have much less severe phenotypes does provide evidence for a misinterpretation of data by DiStefano et al (Cell Stem Cell, 2019). This is not surprising as it is known that P body disintegration can result through multiple mechanisms and is consequence rather than cause of the primary defect. For example, see <https://www.ncbi.nlm.nih.gov/pmc/articles/PMC1900022/>.
: Yes, there are several reports confirming that P-body disruption does not disable gene repression activities. However, we do not think that P-bodies are just a by-product of these regulatory activities because KO of *Eif4enif1* and *Dcp2*, whose functions are known to be directly related to P-body metabolism, also altered the transcriptome. We added more on this in the Discussion.

Major issues:
1) Differential expression analysis using only two samples of each genotype is statistically unsound, especially given the huge variability between the two samples. Also, given that wt and mutant embryos are so strikingly different by E8.5, it is hard to interpret the bulk sequencing. That is, what is a primary defect vs. a secondary defect due to the absence of entire portions of the embryo. Capturing wt vs. mutant earlier when the wt and mutants have yet to show striking phenotypes would be much more interpretable. I appreciate that this could be technically difficult. At minimum, authors should acknowledge limitations and push all embryonic RNA seq analysis to supplement and simply use it to drive follow-up experiments such as stainings and qRT-PCRs. Greater emphasis could be given to DE analysis of the RNA-seq of the EpiLCs as they do not suffer from the issue of secondary defects associated with the morphological defects. However, again, there needs to be a minimum of 3 samples for each genotype to do such analysis.

: According to the suggestion, we moved E8.5 embryo RNA-seq data to the supplement and conducted more whole-mount ISH analyses using E7.5 embryos and DEG analyses using the four KO ESC RNA-seq data. Regarding the sample number for cell RNA-seq, as we had many different conditions to check (WT, *Ddx6* KO*, Dgcr8* KO*, Eif4enif1* KO*, Dcp2* KO in both ESC & EpiLC Day2 stage), we wanted to reduce the library number if possible. As we noted that ESCs do not exhibit large variance in gene expression on qRT-PCR, we considered n = 2 sufficient for the ESC stage. We checked the reproducibility between two ESC libraries of each KO and it was good. In the case of EpiLC, many differences appeared during the induction process, i.e., the freshness of growth factors, etc., so we used n = 3.

2) Given the most significant conclusion the authors make is diminished BMP and excess Nodal signaling, they should directly measure those signaling pathways using relevant phosphoSMAD antibodies including IF staining of embryos and Westerns of EpiLCs.
: Unfortunately, the available antibody did not work well for IF staining on the embryo section. Thus, we were unable to provide IF images of *Ddx6* KO embryos with pSMADs, but we confirmed a decrease in p-SMAD1/5/8 in mESCs by Western blot assay (shown in Fig. 4J). However, different from our expectation of enhanced Nodal signaling, the p-SMAD2 level did not change (Fig. S4A). We explained this point in the Discussion.

3) Either a more thorough characterization of DGCR8 embryos should be performed or any statements of phenotypic similarity should be guarded. For example do DGCR8 embryos show similar defects in the domains of Nodal and EOMES, premature SOX1, somite morphogenesis, etc. Given that the similarity is a major conclusion of the paper, a more thorough characterization is preferred.
: As described above, we prepared new samples and characterized *Dgcr8* KO embryos via WISH with *Brachyury, Nodal*, and *Eomes* probes. As *Dgcr8* KO embryos were markedly malformed at E8.5, we chose E7.5 to compare with *Ddx6* KO phenotypes.

4) Figure panels are poorly organized and labeled (e.g. all embryo panels should make clear the developmental stage). Many of the images are rather low quality, although the phenotype is typically clear regardless. Still, it would be nice to get better images for publication. Also need to provide quantification such as how many embryos were analyzed and shows particular phenotype including morphological and staining defects.

: We added new images and all requested information.

5) Analysis in Fig. 7F and G is confusing and superficial. It should be removed or thoroughly followed up.
: They were removed.

6) Given that the paper is essentially a DDX6 paper, the introduction should provide more background on DDX6 including previous biochemical work studying the molecular regulation of mRNA stability/ translational regulation by DDX6.
: We supplemented this part.

7) Conclusion is confusing and often tangential. Should focus on main points of paper: Points 1 through 3 under general comments above. For example the extensive discussion of DDX6 roles on the ISG pathway comes out of nowhere and seems irrelevant. Also, the discussion of Fig. 7F and G should be removed along with the data unless a much more thorough analysis (i.e. follow-up experiments) is done, which does not seem realistic.

: We revised the Discussion section in a way that focuses on the findings of our study. We mainly discussed BMP/ Nodal signaling and the results of genetic dissection of DDX6 pathways.

▶ We provide the tokens for our RNA-seq data so that reviewers can access.

czobcaqmhpcrhax for GSE171156 (E8.5 embryo RNA-seq)

ujyfkekypncxhgb	for GSE187390 (ESC & EpiLC RNA-seq)

We also provide the numerical data of qPCR analyses as a supplementary file.

**[Reviewer #2]**

Major comments:
When comparing to Di Stefano et al, it seems that another difference is the acute depletion of DDX6 via siRNA or CRISPRi versus longer term knockouts in this work. This might be included in the discussion as another potential source of disagreement between the two studies – that acute versus prolonged DDX6 depletion may differ.
: We were also careful about such acute vs. prolonged effects of DDX6 depletion. Thus, one of the main purposes of the *Ddx6* cKO (Fig. 5) was to confirm that upregulation of *Nodal* and the negative regulators of BMP signaling occurs immediately after the depletion of DDX6, and the result was positive. Moreover, the *Ddx6*-deficient cell phenotype itself was similar between *DDX6* KD hESCs and our KO mESCs. Both exhibited high *Nanog* expression and were unable to exit ground state pluripotency.

Adding a rescue experiment to Figure 5 where DDX6 is restored to knockout cells using a transgene would be a strong demonstration that the phenotypes in this work are specific to DDX6. Without this, the possibility remains that some unknown factor is mediating the difference between the conclusions in this work and prior work.
: We understand the reviewer’s comment. As answered above, however, we think that the observed *Ddx6*-deficient phenotype itself is consistent with that described by Di Stefano et al. What we claim to be different is the interpretation of the dependency of the DDX6 function on P-bodies. Di Stefano et al. depleted LSM14A, one of the core P-body components, to disrupt P-body assembly then noted that it phenocopied *DDX6* KD. Therefore, they concluded that DDX6 function is P-body mediated. However, when we deleted *Eif4enif1*, another core P-body component, P-bodies disappeared but it did not phenocopy *Ddx6* KO. Therefore, we suggest that DDX6 function in pluripotent cells is not dependent on P-bodies, but rather the DDX6-LSM14A complex has an essential role in gene expression regulation. In addition, as our *Ddx6* KO mESCs phenotypes are similar to those of *DDX6* KD hESCs by Di Stefano et al., (hyper-pluripotent with high *Nanog* expression) and *Ddx6* KO mESCs by Freimer et al., (lower proliferation rate and more dispersed morphology of the colony), we do not think that it is necessary to provide extra confirmation of whether this is truly the result of the loss of DDX6.

Minor comments:
Is Fig1a E7.5 panel a composite image of multiple images? If so, please separate the individual images by whitespace.

: We exchanged the images.

**[Reviewer #3]**

1. Line 167 “…genes of major developmental processes, especially the formation of mesoderm derivatives, were downregulated in Ddx6 KO (Fig. 1B)”: Not clear which GO categories are referred to. Examples would help.

: The GO terms related to the formation of mesoderm derivatives are: muscle tissue development, muscle organ morphogenesis, cardiac myofibril assembly, skeletal muscle organ development, and connective tissue development. We added this detailed list in the main text.

2. As is, evidence for decreased BMP signaling is mainly consequential or via RNA. Protein evidence such as through staining for P-SMAD159 (see e.g. Senft et al 2019 Nature Communications, PMID: 30842446) would help strengthen the instructive role of BMP signaling in the observed Ddx6 KO phenotype.
: Please see our response to Reviewer #1’s second major comment. We obtained evidence of suppressed BMP signaling by conducting Western blot analysis (shown in Fig. 4J).

3. Embryo stage should be included in ALL panels (e.g. Figure 3A, D, E and other figures)
: We added all information.

4. Line 240: Reference to panels D,E missing.
: Added.

5. Figure 5H: The way the data is plotted does could either suggest SMAD1/5 targets are de-repressed in Ddx6 KOs (as suggested by the authors) or alternatively that these genes are not repressed in Ddx6 KOs upon differentiation. A plot such as in 5D showing both WT and KO levels would clarify this.
: We changed the graph format to 5D (Fig. 4D in the revised manuscript) and confirmed that BMP-SMAD targets are expressed highly from the ESC stage. A large difference was observed on EpiLC Day1, which is consistent with their role as early differentiation regulators, and their expression levels decreased similar to those in WT on EpiLC Day2.

6. RNA-seq (or other type of) validation of successful KO of Eif4enif1 and Dcp2 should be included.

: We confirmed successful targeting by checking each gene locus using the Integrative Genomics Viewer (IGV) (Fig. S5C).

7. Lines 456-458: This section of how putative BMP inhibitor regulating TFs were categorized would be useful in the respective results section and then discussed as is.

: Following Reviewer#1’s suggestion, we removed this analysis.
