# Supplementary material for: The RNA helicase DDX6 controls early mouse embryogenesis by repressing aberrant inhibition of BMP signaling through miRNA-mediated gene silencing
Source: PLoS Genet. 2022 Oct 5;18(10):e1009967. doi: 10.1371/journal.pgen.1009967 (PMC9534413; doi:10.1371/journal.pgen.1009967)
Supplement: Attachment — Submitted filename: Response to reviewers-editor revision.docx [file pgen.1009967.s013.docx]

Responses to reviewer’s comments

**[Reviewer #2]**

Reviewer #2: My one comment that would improve the paper in its current version is to add a discussion of other reasons why Dcp2 and Eif4enif1 might differ from DDX6 beyond just their role in P-body formation, for example because they have molecular functions of their own that are different than DDX6’s. It is very hard to assign a function specifically to a biomolecular condensate as opposed to a molecular function of the entities that form a biomolecular condensate. DDX6, Eif4enif1, and Dcp2 are in P-bodies and also all have independent and distinct molecular functions, so differentiating molecular functions from functions of biomolecular condensates requires more than knockouts. This could be included in the discussion.

: When introducing our genetic dissection model in the previous manuscript, we briefly explained individual protein’s molecular function, but it seemed that it was too brief. Thus, this time we wrote a more detailed introduction for each protein (EIF4ENIF1 (4E-T), DCP2, and DGCR8). We tried to make a simplified model to simultaneously examine the DDX6-associated molecular pathways using several KO ES lines, but it is indeed still complicated. Because of the nature of DDX6 being highly related to P-bodies, its interacting partners 4E-T, DCP2, and the miRNA pathway are all related to P-body morphology/functions. We acknowledge that via this genetic approach, we cannot precisely identify molecular functions of the biomolecular condensate, P-bodies, and even each protein. Instead, we have further analyzed the GSEA results to see which gene sets or cellular processes were disrupted when a certain RNA-regulatory pathway was nonfunctional. We re-categorized the GSEA results to provide the gene sets that are characteristically affected in three different conditions: miRNA pathway-related but P-body-independent (*Ddx6* KO & *Dgcr8* KO only)/ miRNA-related & P-body-dependent (all four mutants)/ not related to miRNA but P-body-dependent (*Eif4enif1* KO & *Dcp2* KO) (Table 2). We also re-drew the Venn diagram comparing gene expression changes of four mutant ESCs so that the readers can easily recognize the number of gene sets that are associated with the disruption of certain RNA-regulatory mechanisms (Fig. 6B). Another interesting information that is newly added this time is the list of gene sets that are changed only in each KO (Table S3-5). Based on these data, we can assume P-body-independent cellular functions of each protein.

DDX6 and 4E-T or DDX6 and DCP2, they are not the essential co-factors. They are just one of the interactors forming one type of protein complex, so we think it is natural that they have functions that are not related to DDX6. What we have confirmed in this study is that DDX6 mainly works as a component of the miRNA-mediated gene silencing pathway during early embryogenesis, and the degree of involvement of DDX6 in 4E-T-mediated translational repression or in 5’-to-3’ mRNA decay pathway (in relation to DCP2) is little. We interpret that very different phenotypes reflect low correlation/interaction between DDX6 and 4E-T or DCP2. That is the reason why we think it is highly likely that DDX6-LSM14A complex is the main mediator of miRNA-mediated translational repression in early embryos.

We think our genetic analyses can provide an inductive insight on the functions of each RNA-regulating protein and P-bodies. We have described our reasoning underlying the design of the genetic dissection model and the interpretation of the transcriptomic analyses in detail. We hope this change clarifies our thoughts.

**[Reviewer #3]**

- Fig. 5H would be more legible if split into 4 plots (one per mRNA)
- “brackets” used for indicating significance on various plots (e.g. Fig. 4H) are stylistically distracting/unnecessary, asterisks above the compared timepoints would suffice

: I think you meant 4H, not 5H. Fig. 4H and other plots have been changed accordingly.
